# Predictive value of the augmentation index derived vascular age in patients with newly diagnosed atherosclerosis

**DOI:** 10.1007/s00380-016-0868-0

**Published:** 2016-07-11

**Authors:** Stefan Betge, Daniel Kretzschmar, Hans-Reiner Figulla, Michael Lichtenauer, Christian Jung

**Affiliations:** 1 Department of Cardiology, Clinic of Internal Medicine I, Universitätsherzzentrum Thüringen, Friedrich Schiller University Jena, Erlanger Allee 101, 07747 Jena, Germany; 2 Department of Cardiology, Clinic of Internal Medicine II, Paracelsus Medical University of Salzburg, Muellner Hauptstraße 48, Salzburg, 5020 Austria; 3 Division of Cardiology, Pulmonology, and Vascular Medicine, Medical Faculty, University Duesseldorf, Moorenstraße 5, Duesseldorf, 40225 Germany

**Keywords:** Atherosclerosis screening, Augmentation index, Photoplethysmography, Vascular age

## Abstract

Early detection of atherosclerosis, i.e., in occupational health screening programs could reduce the rate of cardiovascular events in the working population. Changes of the augmentation index (AIX) correlate with changes of the arterial stiffness induced by aging, atherosclerosis, or arterial hypertension and have a prognostic value for cardiovascular events. Their diagnostic yield should be increased by normalizing the AIX to age, in terms of a calculating the vascular age (VA). In this pilot study, 30 patients (mean age 65.3 ± 8.8 years, 21 male) with suspected coronary heart disease underwent a duplex ultrasound of the carotid arteries and a measurement of the ankle brachial index in addition to the coronary angiography. The AIX was recorded with a portable device (Vascular Explorer), and the VA was calculated. Atherosclerosis was found in 24 patients. They were older than the patients without atherosclerosis, but there was no age dependency found for the distribution pattern or severity of atherosclerosis. In patients with findings of atherosclerosis, the calculated VA was higher than the chronological age, and these differences were significant in patients below 65 years of age. Comparing patients in higher blood pressure classes with patients in lower classes, significantly higher AIX, VA, and differences to the chronological age were found. The VA, deduced from the noninvasively obtained AIX, is a promising candidate for screening programs for atherosclerosis, i.e., in occupational health screening programs.

## Introduction

Risk assessment for atherosclerosis is an important step in primary prevention of cardiovascular events to identify patients that should undergo more specific diagnostic strategies and medical interventions [1]. Risk assessment models, such as the Framingham score or the European SCORE system, integrate several risk factors for fatal cardiovascular events, including hyperlipoproteinemia, hypertension, gender, and chronological age [2, 3]. However, they provide only a rough estimation of the individual risk of single person [4].

Since the spatial resolution of ultrasound probes was high enough to directly visualize early stages of atherosclerosis, such as changes of the intima media thickness (IMT) in the carotid arteries, and age-dependent normal values had been described, individual values could be normalized to the chronological age. This led to the term “vascular age” [5, 6]. The IMT is an independent predictor of cardiovascular events [7]; however, its main disadvantage is the need of experienced investigators and high-frequency ultrasound probes for accurate measurements. In consequence, its determination is not feasible for screening programs, i.e., in occupational health screening programs.

The concept of the vascular age (VA), however, has been adapted to the Framingham data and the European SCORE system [8–11], as well as regional differences have been implemented into the SCORE system to increase their sensitivity [12, 13], but still there is a need for screening devices with higher predictive value.

The pulse wave velocity (PWV) and the changes in the pulse wave reflections, quantified as augmentation index (AIX), are parameters of arterial stiffness [14]. Pathologic values of these parameters have a prognostic value for mortality and cardiovascular events in symptomatic and asymptomatic patients [15–17]. In a recently published twin study, the age dependency of these parameters has been confirmed as well as the genetic influence [18]. Changes in the arterial stiffness are additionally the result of influences, such as arterial hypertension and atherosclerosis. There are several devices available for the noninvasive measurement of the PWV and AIX.

The aim of this pilot study was to measure the PWV and AIX with the photoplethysmography-based Vascular Explorer (Enverdis, Germany) [19] and to evaluate the concept of normalizing these parameters to age-dependent normal values, resulting in a new variation of the term vascular age. This normalization should increase the predictive value of these parameters concerning the presence or absence of atherosclerosis, i.e., in the setting of occupational health screening programs.

## Methods

### Patients and screening procedures

All subsequent patients, who were submitted in our department with suspicion for coronary heart disease for a heart catheterization between November 2010 and February 2011, were screened and patients with known atherosclerotic disease at any site excluded. After written informed consent, the screening for atherosclerosis was performed with duplex ultrasound examination of the extracranial cerebral arteries, a measurement of the ankle brachial index (ABI) in rest and after standardized exercise test and angiography of the coronary arteries. Arteriosclerotic disease was quantified in the extracerebral and coronary arteries as no atherosclerosis (0 points), irregularities or thickening of the vessel wall (1 point), and plaques or stenoses (2 points). Peripheral atherosclerosis was quantified as normal ABI in rest and after exercise (0 point) or pathologic values of the ABI either in rest or in only after exercise (2 points).

Blood pressure measurements were performed in every patient in three different situations: (1) during the measurements with the Vascular Explorer device in the early morning in rest, (2) by the nurses on the ward during the morning, and (3) right before performing the exercise ECG. The blood pressure regulation was classified according to the classification of the European Society of Cardiology [20].

### Photoplethysmographic measurements

The pulse wave velocities and the augmentation indices as parameters for arterial stiffness were measured with the Vascular Explorer (Enverdis, Jena, Germany). Measurements were performed after 5 min of rest in a quiet and temperature-controlled room in supine position. Blood pressure measurements and pulse wave analysis were performed with photoplethysmographic sensors and inflatable upper arm and lower leg cuffs, registering the volume changes in the cuffs due to the pressure changes in the underlying arteries by the pulse waves, as described in detail elsewhere [21]. The ankle brachial indices (ABI) were calculated using the blood pressure values in the lower and upper extremities. The PWV and the AIX for the pressure curves in the central aorta were calculated with specific transfer functions [22, 23]. As a new function, the PWV and AIX for the central aorta were normalized to age-dependent normal values, resulting in the VA. The age-dependent normal values for the PWVao and AIXao were calculated out of the data of the Anglo-Cardiff Collaborative Trial (ACCT) [24]. In a final step, the differences of the VA to the chronological age were calculated and compared between the groups.

The VA was additionally determined via the charts of the European SCORE system [10], and differences to the chronological age were calculated.

The study was approved by the local ethics committee of the University of Jena (reference number 2691-11). All procedures were performed in accordance with the recommendations in the Helsinki Declaration. ClinicalTrial.gov identifier: NCT01091194.

### Statistical analysis

Continuous data are expressed as mean ± SD, and categorical data are presented as counts and percentages. All data were analyzed using IBM^®^ SPSS^®^ Statistics Version 20 (IBM Corporation). Categorical data *χ* ^2^ and Fisher’s exact test were used. The Mann–Whitney *U* test was used for between-group comparisons. Statistical significance was assumed at a *P* < 0.05.

## Results

### Patients characteristics

Thirty patients (21 male, 9 female) with a mean age of 65.3 ± 8.8 years were included into this study. The baseline characteristics are summarized in Table 1. The diagnosis hyperlipoproteinemia was found in the list of diagnosis of 18 patients; only 3 of these patients were on statin therapy at the time of admission; all of them had a hyperlipoproteinemia as defined in our study protocol as total cholesterol >5 mmol/l and/or LDL >3 mmol/l. In the group of the other 12 patients without a former diagnosis of hyperlipoproteinemia, 8 patients were on statin therapy at the time of admission and 3 had high levels of total cholesterol and/or HDL measured. Of the 21 patients with measured hyperlipoproteinemia at time of admission, 5 patients were on statin therapy.

**Table 1:** Demographic data of patients with or without atherosclerosis of any degree and any localization

	No atherosclerosis (*n* = 6)	Atherosclerosis (*n* = 24)	*p* value
Sex(% men)	66.7	70.8	1.0
RR Class (% ≤ 3)	66.7	37.5	0.360
BMI (kg/m^2^)	28.0 ± 5.7	28.9 ± 3.4	0.743*
Active smoker (*n*)	0	3	1.0
Former smoker (*n*)	1	6	1.0
Diabetes mellitus (*n*)	0	6	0.302
Cholesterol >5 mmol/l (*n*)	5	15	0.633
LDL >3 mmol/l (*n*)	4	14	1.0
Statins on admission (*n*)	1	10	0.372
C-reactive protein (mg/l)	2.3 ± 2.2	5.2 ± 10.3	0.218
Erythrocyte sedimentation (1 h, mm)	10.7 ± 9.2	18.6 ± 16.7	0.145
Erythrocyte sedimentation (2 h, mm)	20.5 ± 13.6	36.5 ± 22.3	0.046
White blood cell count (mcL)	8.1 ± 2.4	7.3 ± 1.7	0.476

RR class, classification of the blood pressure regulation according to the classification of the European Society of Cardiology [20]. *BMI* body mass index, *LDL* low density lipoprotein

* Mann–Whitney *U* test

Of the 6 patients with diabetes mellitus type 2, two patients were on insulin therapy. The 3 active smokers in our study had an acquired burden of 21.7 ± 7.7 pack-years, and the 7 former smokers (stopped since 23.4 ± 19.5 years) had an acquired burden of 16.9 ± 10.2 pack years (Table 1).

Twenty-two of the 30 patients had been on antihypertensive drugs due to known arterial hypertension; 14 patients were on single drug treatment (8 on beta blockers, 2 on ACE-Inhibitors, and 4 on sartans), 6 patients on a combination of beta blockers and ACE-Inhibitors, and 5 patients on a combination of beta blockers and sartans. The mean values of the AIX were not different between the patients without or with regular intake of ACE inhibitors or sartans (AIXao 33.8 ± 11.8 vs. 33.2 ± 11.5); however, lower values were measured in the patients with high daily doses of the substances, i.e., 40 mg Enalapril, 10 mg Ramipril, 32 mg Candesartan (AIXao 25.3 ± 10.1). The same tendency—to a lower extent—was seen in the patients on beta-blockers. Due to low numbers of patients, the differences were not statistically significant.

After serial measurements of the blood pressure during the hospital stay, high blood pressure values were found in all patients, 13 patients were classified into the blood pressure classes 1, 2, or 3 and another 17 patients in the blood pressure classes 4 and 5.

The white blood cell counts ranged from 5.000 to 12.700/mcL (mean 7.483 ± 1.860/mcL), the erythrocyte sedimentation from 1 to 81 mm/h (mean 17.3 ± 15.8 mm/h), and the concentration of C-reactive protein from below the detection threshold of 2.5–48 mg/l (mean 4.6 ± 9.2 mg/l). Erythrocyte sedimentation after 2 h differed significantly between patients without and with atherosclerosis (*p* = 0.046). The concentration of C-reactive protein tended to be higher in patients with atherosclerosis, however, likely due to the small size of our patient cohort; no statistically significant difference was found. AIX did also not correlate with the aforementioned markers of inflammation.

Atherosclerosis of any degree was found in 24 of the 30 patients. These were 81.0 % of the male patients and 77 % of the female patients.

In the analysis of the atherosclerotic pattern in the single patients, there was no age dependency found (Table 2), but the patients with atherosclerotic disease were significantly older compared with the patients without atherosclerotic lesions at any site (Table 3). Every patient above the age of 65 years had a positive finding of atherosclerosis at any localization or of any degree.

**Table 2:** Degree and localization of atherosclerosis of all patients, sorted according to the chronological age

Age	Extracranial cerebral artery disease	Coronary artery disease	Peripheral arterial disease	Sum of points	Arterial disease of any degree
43.6	0	0	0	0	No
51.2	0	0	***2***	2	Yes
52.4	0	0	0	0	No
55.2	0	0	0	0	No
58.2	0	*1*	0	1	Yes
58.2	0	*1*	0	1	Yes
59.2	0	0	0	0	No
59.2	**2**	**2**	0	4	Yes
59.2	*1*	**2**	***2***	5	Yes
59.4	**2**	**2**	0	4	Yes
61.4	**2**	0	0	2	Yes
62.5	**2**	0	***2***	4	Yes
62.9	0	0	0	0	No
63.0	0	0	0	0	No
63.5	*1*	**2**	0	3	Yes
64.7	**2**	**2**	***2***	6	Yes
68.4	0	1	0	1	Yes
69.5	*1*	**2**	0	3	Yes
70.1	0	**2**	0	2	Yes
70.6	0	*1*	0	1	Yes
71.2	**2**	**2**	0	4	Yes
72.3	0	**2**	0	2	Yes
72.3	*1*	**2**	0	3	Yes
73.3	0	**2**	0	2	Yes
74.8	*1*	**2**	0	3	Yes
75.0	0	**2**	0	2	Yes
76.9	0	*1*	0	1	Yes
77.0	0	**2**	0	2	Yes
77.0	**2**	*1*	0	3	Yes
77.3	*1*	**2**	***2***	5	Yes

Italic values: irregularities or thickening of the vessel wall (1 point). Bold values: plaques or stenoses (2 points). bold–italic values: pathologic ABI measurement (2 points). RR class, classification of the blood pressure regulation according to the classification of the European Society of Cardiology [20]

**Table 3:** Results of the measurements with the Vascular Explorer and calculations of the vascular age using several formulas in patients with or without atherosclerosis at any localization and of any degree

	No atherosclerosis (*n* = 6)	Atherosclerosis (*n* = 24)	*p* value
Age	56.0 ± 7.4	67.6 ± 7.6	0.006
PWVao	7.84 ± 0.88	8.60 ± 1.55	0.173
AIXao	24.00 ± 7.04	35.83 ± 10.45	0.021
VA-ESC	69.5 ± 11.55	80.38 ± 7.26	0.025
VA-PWVao	56.29 ± 7.63	64.7 ± 10.1	0.049
VA-AIXao	54.73 ± 8.18	82.86 ± 21.40	0.012

*PWVao*, pulse wave velocity in the central aorta, *AIXao*, augmentation index in the central aorta, *VA-ESC*, vascular age determined via the charts of the European SCORE system [10], *VA-PWVao*, vascular age calculated from the PWVao and the data of the ACCT [24], *VA-AIXao*, vascular age calculated from the AIXao and the data of the ACCT [24]

Atherosclerosis of any degree was found in 69.2 % of the patients with arterial hypertension in the classes 1–3 and in 88.2 % of the patients with arterial hypertension in the classes 4 and 5 (*p* = 0.36).

### Augmentation index, pulse wave velocity, and vascular age

**Fig. 1:**
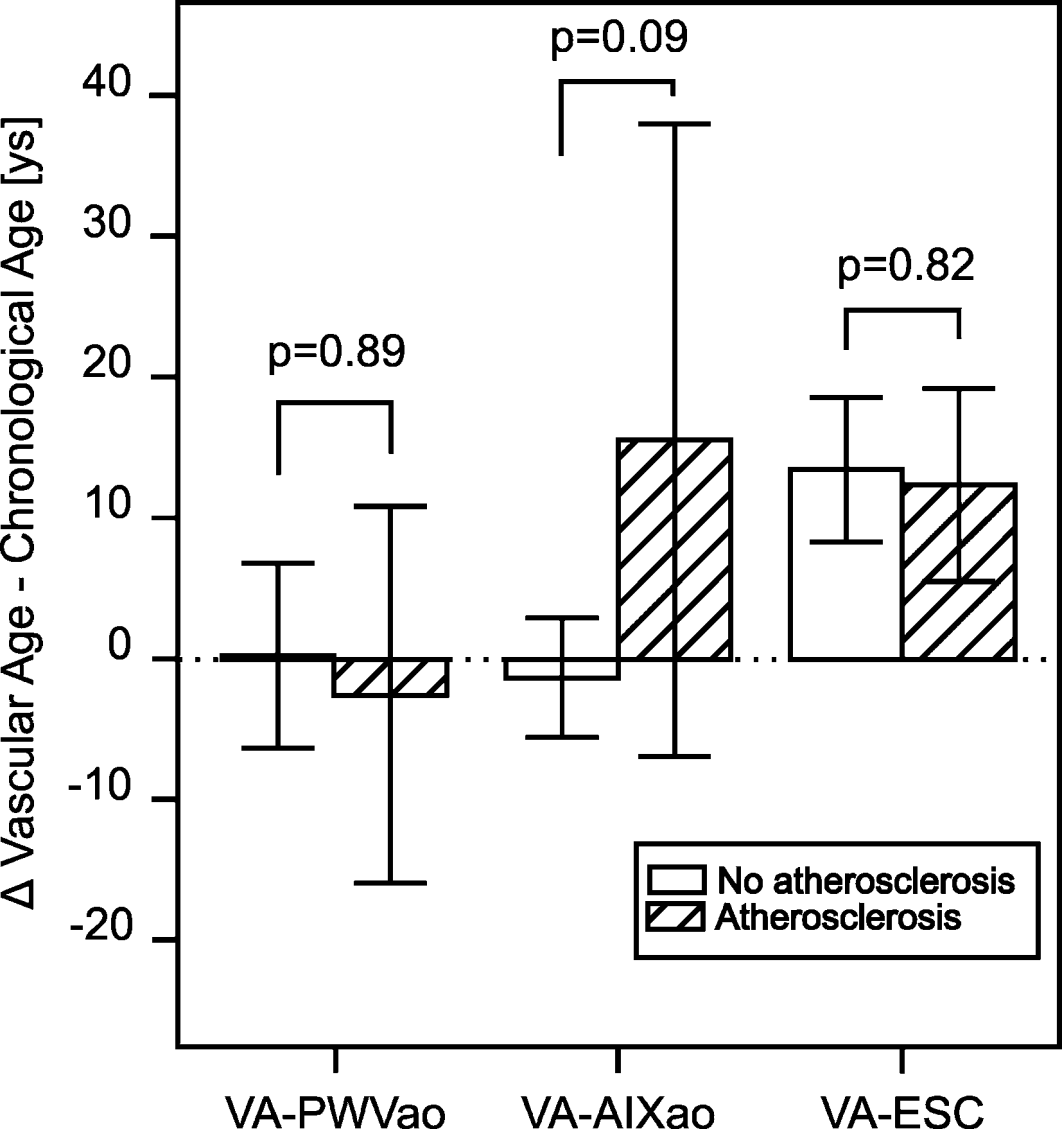
Differences between the patients’ chronological age and the calculated vascular ages in years (ys) in patients with or without atherosclerosis. VA-PWVao, vascular age calculated from the PWVao and the data of the ACCT [24]. *VA-AIXao*, vascular age calculated from the AIXao and the data of the ACCT [24]. *VA-ESC*, vascular age determined via the charts of the European SCORE system [10]

The underlying thesis of this study was that the normalization of the measurements of PWV and AIX to the age-dependent normal values—resulting in a new variation of the parameter vascular age—and calculating the difference between this vascular age (VA) and the chronological age (CA) can discriminate between patients with atherosclerosis and no atherosclerosis. The measured values for the PWV and AIX and the deduced VA are shown in Table 3, resulting differences between the vascular age and the chronological age (ΔVA–CA) in Fig. 1. Patients with findings of atherosclerosis were significantly older, as were the calculated VA in our study population (Table 3).

As the main finding of our pilot study, we found the vascular ages, deduced from the AIXao (VA-AIXao), in the same range as the CA in the patients without atherosclerosis (mean Δ VA-CA −1.3 ± 4.2 years), while they were higher in the group of patients with atherosclerosis (mean ΔVA–CA +23.8 ± 19.6 years). This difference between the groups was not significant (*p* = 0.09) (Fig. 1).

**Fig. 2:**
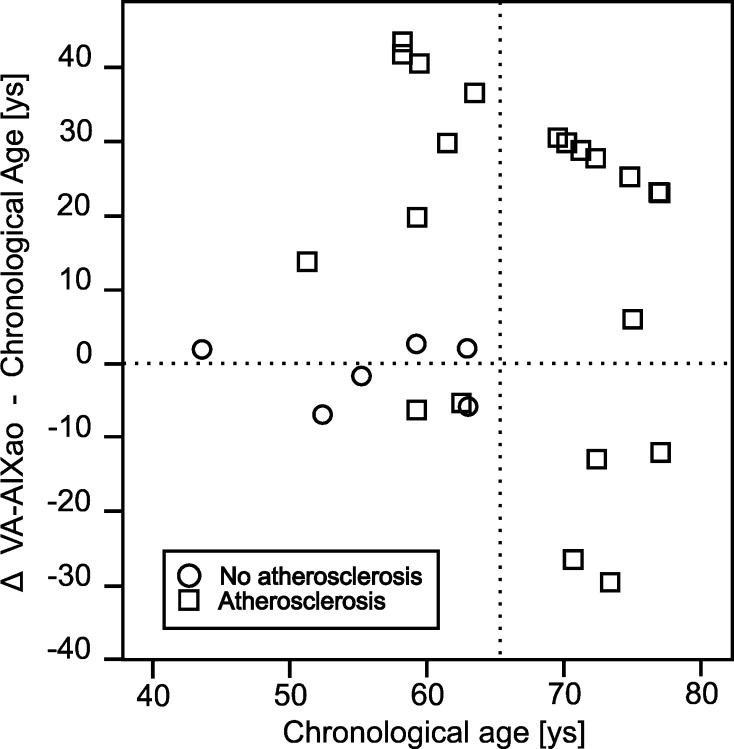
Differences between the calculated vascular age (VA-AIXao) and the patients’ chronological age, plotted against the chronological age in years in patients with or without atherosclerosis. VA-AIXao, vascular age calculated from the AIXao and the data of the ACCT [24]

To discriminate if there are age-dependent differences in the accuracy of the parameter, we plotted the ΔVA–CA for every single patient against the CA: in the patients above 65 years, the parameter VA-AIXao did not reflect the vascular status of the patients: the ΔVA–CA were dispersed between −29.6 and +30.5 years, although all these patients had atherosclerotic findings. This was different for the patients below 65 years: in those with atherosclerosis (mean age 59.8 ± 3.8 years)—except two—the mean calculated VA-AIXao was with 83.0 ± 20.0 significantly higher than the CA, while in the patients without atherosclerosis (mean age 56.0 ± 7.4 years), the VA was on the same level (mean VA-AIXao 54.7 ± 8.2) of the CA (Fig. 2). Comparing the ΔVAao-CA of the patients <65 years with atherosclerosis with these of the patients without atherosclerosis, a *p* value of *p* = 0.036 was calculated.

The other variations of the vascular age (VA-PWVao and VA-ESC) did not differentiate between the patient groups.

**Fig. 3:**
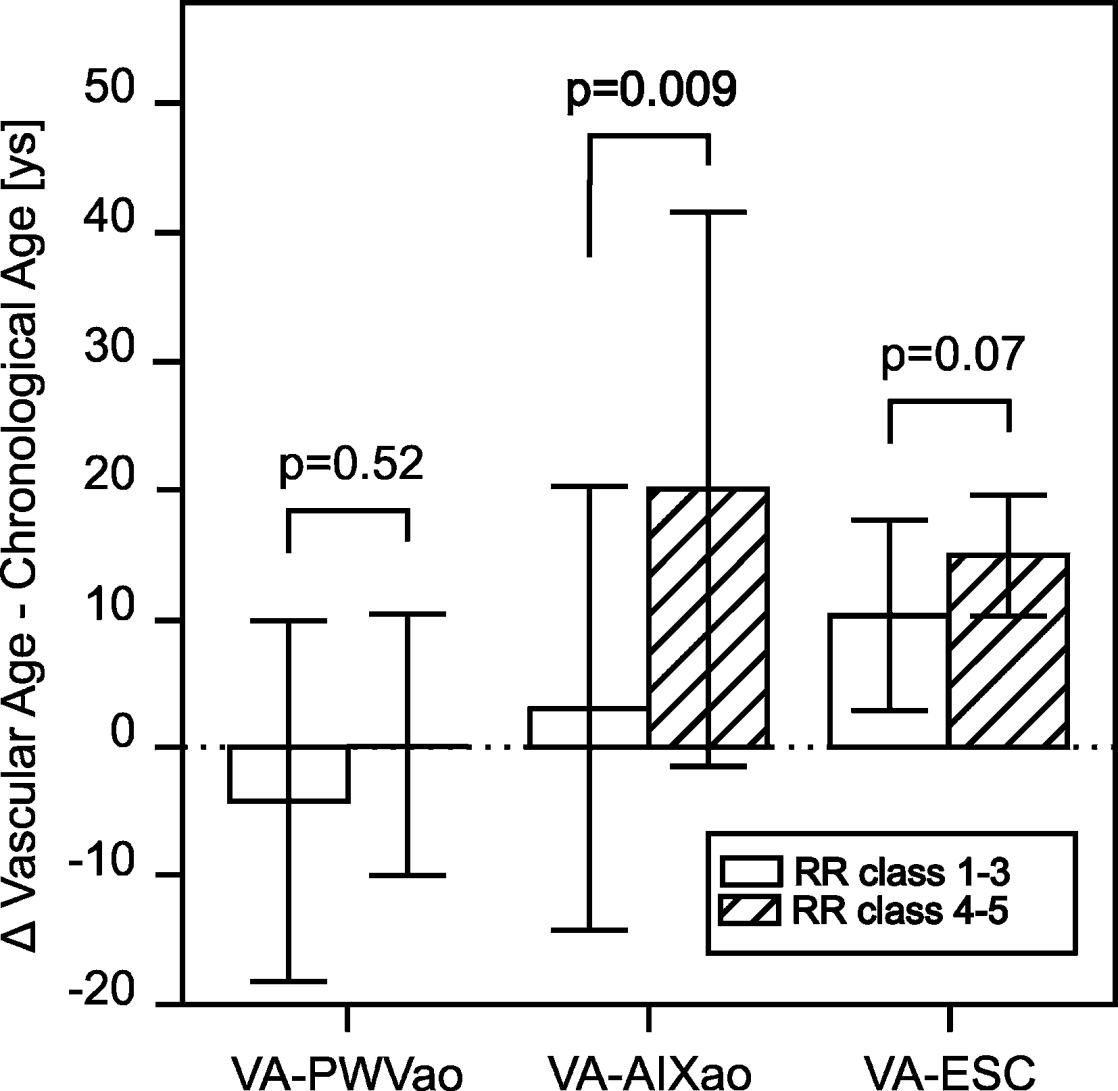
Differences between the patients’ chronological age and the calculated vascular ages in years in patients in lower or higher blood pressure classes. VA-PWVao, vascular age calculated from the PWVao and the data of the ACCT [24]. *VA-AIXao*, vascular age calculated from the AIXao and the data of the ACCT [24]. *VA-ESC*, vascular age determined via the charts of the European SCORE system [10]

The same comparisons had been made for the patients in lower vs. higher RR classes. The CA of the patients did not differ comparing the groups, but we did find significantly higher augmentation indices (AIXao) and pulse wave velocities (PWVao), as well as higher vascular ages in the patients with higher blood pressure values (Table 4). The VA-AIXao were found in the same range as the CA in the patients in lower RR classes (mean ΔVA–CA +3.0 ± 17.2 years), while they were significantly higher in the group of patients in the higher RR classes (mean ΔVA–CA +20.0 ± 21.5 years) (Fig. 3).

**Table 4:** Results of the measurements with the Vascular Explorer and calculations of the vascular age using several formulas in patients with RR Classes ≤3 or ≥4

	RR classes ≤3 (*n* = 13)	RR Classes ≥4 (*n* = 17)	*p* value
PWVao	8.03 ± 1.21	8.79 ± 1.60	0.02
AIXao	26.92 ± 7.65	38.47 ± 10.45	0.002
Age	63.0 ± 11.0	67.07 ± 8.78	0.363
VA-ESC	73.23 ± 10.66	82.0 ± 5.68	0.015
VA-PWVao	58.83 ± 10.61	66.61 ± 8.4	0.061
VA-AIXao	66.01 ± 22.18	86.45 ± 20.53	0.017

RR classes, classification of the blood pressure regulation according to the classification of the European Society of Cardiology [20]. *PWVao*, pulse wave velocity in the central aorta, *AIXao*, augmentation index in the central aorta, *VA-ESC*, vascular age determined via the charts of the European SCORE system {}. *VA-PWVao*, vascular age calculated from the PWVao and the data of the ACCT [24]. *VA-AIXao*, vascular age calculated from the AIXao and the data of the ACCT [24]

## Discussion

In our pilot study, the calculation of the vascular age by normalization of the augmentation index AIXao with age-dependent normal values allowed the screening for vascular changes due to arterial hypertension or atherosclerotic changes, for the latter with the highest diagnostic yield in individuals at or below the age of 65 years. Since both, atherosclerosis and high blood pressure increase the cardiovascular risk and these measurements can be done noninvasively with portable devices, this screening method seems to be suitable for screening programs for primary prevention of cardiovascular events, i.e., in occupational health screening programs.

It has been shown that the intake of beta blockers, ACE inhibitors, and angiotensin-1-receptor-antagonists reduces the measured augmentation index in patients with stages I and II arterial hypertension as well as in a variety of other indications [25, 26]. These effects could be shown in our data concerning high doses of ACE inhibitors and angiotensin-receptor antagonists. However, the most effective reduction of the AIX has been recorded when the therapeutic goal of low blood pressure values had been reached.

In our study, the measurements of the PWV and AIX, as well as the calculation of the VA, were done with the Vascular Explorer. In a study comparing the Vascular Explorer with the SphygmoCor (Atcor Medical, Sydney, Australia) and the Arteriograph (TensioMed, Budapest, Hungary), two other devices for plethysmographic measurements of PWV and AIX, there were no significant differences concerning the values of the measured PWV in repetitive measurements in 44 individuals. The AIX was measured at a higher level with the SphygmoCor compared to Arteriograph and Vascular Explorer, while the results were comparable among the latter devices. Measurements with the Vascular Explorer gave comparable results in the sitting and supine position [21].

The source of the age-dependent normal values that were used for the calculation of the vascular age in this study had been the database of the Anglo-Cardiff Collaborative Trial (ACCT). In this population study, over 10.000 randomly selected individuals from East Anglia and Wales had been examined. For the determination of the age related changes of the AIX and PWV, the data of individuals with diabetes, arterial hypertension, renal disease, or cardiovascular disease had been excluded. Finally, the data of 4.001 healthy individuals had been analyzed. The PWV and the AIX had been measured in ACCT with the SphygmoCor device in a sitting position; the results were grouped by gender and decade of age. Nearly two-thirds of this study population was between 40 and 69 years old, 14 % were in the eighth decade and only 2 % (77 individuals) in the ninth decade. Consecutively, the calculated normal values for the PWV and AIX for the older people are based on a smaller number of individuals [24].

In our pilot study, the age-dependent normal values of these healthy individuals were used for the first time to our knowledge to calculate the vascular age (VA) in patients with suspicion for coronary heart disease. These patients were extensively screened for findings of atherosclerosis.

The VA derived from the AIXao could discriminate very well between patients with or without atherosclerosis, either in the coronary arteries, the extracranial arteries, or in the peripheral arteries of the lower extremity in patients between 40 and 65 years of age, the typical age for occupational health screening programs.

Our pilot study has some limitations. The most important is the limited number of included patients. Another limitation is the distribution of age and atherosclerotic findings in our study population. All our patients above 65 years of age had atherosclerotic findings in at least one area.

## Conclusions

In summary, we found that in this pilot study, the parameters VA-AIXao have a very promising candidate for screening programs for atherosclerosis, i.e., in occupational health screening programs. This result of our pilot study has to be confirmed in a larger study population.
